# High-resolution spatio-temporal risk mapping for malaria in Namibia: a comprehensive analysis

**DOI:** 10.1186/s12936-024-05103-w

**Published:** 2024-10-05

**Authors:** Song Zhang, Punam Amratia, Tasmin L. Symons, Susan F. Rumisha, Su Yun Kang, Mark Connell, Petrina Uusiku, Stark Katokele, Jerobeam Hamunyela, Nelly Ntusi, Wilma Soroses, Ernest Moyo, Ophilia Lukubwe, Chivimbiso Maponga, Dominic Lucero, Peter W. Gething, Ewan Cameron

**Affiliations:** 1 The Kids Research Institute of Australia, Perth, WA Australia; 2 Curtin University, Bentley, WA Australia; 3 Ifakara Health Institute, Dar es Salaam, Tanzania; 4 National Vector Control Department, Windhoek, Namibia; 5 World Health Organization, Windhoek, Namibia; 6 Clinton Health Access Initiative, Boston, MA USA; 7 National Institute for Medical Research, Dar es Salaam, Tanzania

**Keywords:** Malaria control, Spatio-temporal, Hierarchical model, Risk maps

## Abstract

**Background:**

Namibia, a low malaria transmission country targeting elimination, has made substantial progress in reducing malaria burden through improved case management, widespread indoor residual spraying and distribution of insecticidal nets. The country's diverse landscape includes regions with varying population densities and geographical niches, with the north of the country prone to periodic outbreaks. As Namibia approaches elimination, malaria transmission has clustered into distinct foci, the identification of which is essential for deployment of targeted interventions to attain the southern Africa Elimination Eight Initiative targets by 2030. Geospatial modelling provides an effective mechanism to identify these foci, synthesizing aggregate routinely collected case counts with gridded environmental covariates to downscale case data into high-resolution risk maps.

**Methods:**

This study introduces innovative infectious disease mapping techniques to generate high-resolution spatio-temporal risk maps for malaria in Namibia. A two-stage approach is employed to create maps using statistical Bayesian modelling to combine environmental covariates, population data, and clinical malaria case counts gathered from the routine surveillance system between 2018 and 2021.

**Results:**

A fine-scale spatial endemicity surface was produced for annual average incidence, followed by a spatio-temporal modelling of seasonal fluctuations in weekly incidence and aggregated further to district level. A seasonal profile was inferred across most districts of the country, where cases rose from late December/early January to a peak around early April and then declined rapidly to a low level from July to December. There was a high degree of spatial heterogeneity in incidence, with much higher rates observed in the northern part and some local epidemic occurrence in specific districts sporadically.

**Conclusions:**

While the study acknowledges certain limitations, such as population mobility and incomplete clinical case reporting, it underscores the importance of continuously refining geostatistical techniques to provide timely and accurate support for malaria elimination efforts. The high-resolution spatial risk maps presented in this study have been instrumental in guiding the Namibian Ministry of Health and Social Services in prioritizing and targeting malaria prevention efforts. This two-stage spatio-temporal approach offers a valuable tool for identifying hotspots and monitoring malaria risk patterns, ultimately contributing to the achievement of national and sub-national elimination goals.

**Supplementary Information:**

The online version contains supplementary material available at 10.1186/s12936-024-05103-w.

## Background

Malaria remains a major public health problem worldwide [1, 2]. Namibia is a low malaria transmission country in sub-Saharan Africa, which has successfully reduced malaria burden through coverage of indoor residual spraying, long-lasting insecticidal nets and increased availability of rapid diagnostic tests and effective treatment [3, 4]. As Namibia approaches elimination new approaches are needed to identify and target increasingly isolated transmission foci [5–7]. This work addresses the challenge of hotspot identification, where in this study was referred to a week where incidence rate exceeded 1 case per 1000 person-years-observed (PYO) in each district, providing situational risk maps capable of resolving risk at increasingly high spatio-temporal resolution.

Namibia is one of the eight countries under the Elimination Eight Initiative working across national borders to eliminate malaria in southern Africa by 2030 [6], with its current strategic plan aiming for zero local cases by 2027. Despite the significant declines from 47.50 cases per 1000 population in 2017 to 10.62 in 2021 [7], the northern territories continue to experience periodic outbreaks, especially in recent years [4, 8]. Transmission in this region is unstable and seasonal, typically peaking in April and May. The Namibian landscape is divided into five geographical regions with vast differences in population density across the country, including the Namib Desert in the west stretching along the Namibian coastline, the Great Escarpment, the Central Plateau from north to south, the Kalahari Desert in the east and the Kavango-Zambezi area in north-eastern Namibia. In low transmission settings, infections cluster into spatially- and temporally- distinct foci [9]. An understanding of endemic malaria risk patterns is necessary to inform the design of targeted interventions and focus investigations. Novel approaches for geospatial risk maps aggregated at district level on a weekly basis may assist to detect and prevent persistent focal low levels of transmission and temporal incidence of malaria in a timely manner.

The study outlines an innovative disease mapping approach developed to address this need and present the resulting high resolution spatio-temporal risk maps for malaria in Namibia. In brief, these maps are generated through statistical modelling that combines a suite of environmental covariates and population values—both remotely sensed and themselves modelled—with clinical malaria case counts gathered from local health facilities through passive surveillance systems. Catchment populations attending the nearby health facilities were estimated via an inverse relationship between travel time distance and average facility attendance and fixed through time. A two-stage approach was developed to address the technical challenge of delivering high-resolution risk maps with local risk updates at weekly cadence. First, the long run expected case count at each facility was estimated via a high-resolution spatial risk model, followed by weekly case count updating via a facility-centred, spatio-temporal sub-model. This approach identifies substantial geographical variation and seasonality in malaria risk in Namibia.

The high-resolution risk maps presented here have been used by Namibian Ministry of Health and Social Services—National Vector-Borne Diseases Control Programme (NVDCP) for malaria stratification to set priorities and target prevention efforts to the areas where it is most needed [10]. We hope that the methods developed in this study will prove useful in similar low transmission settings where risk mapping using weekly case data can provide dynamic and accurate support for the detection, management and control of disease outbreaks.

## Methods

### Response data and covariates

**Fig. 1:**
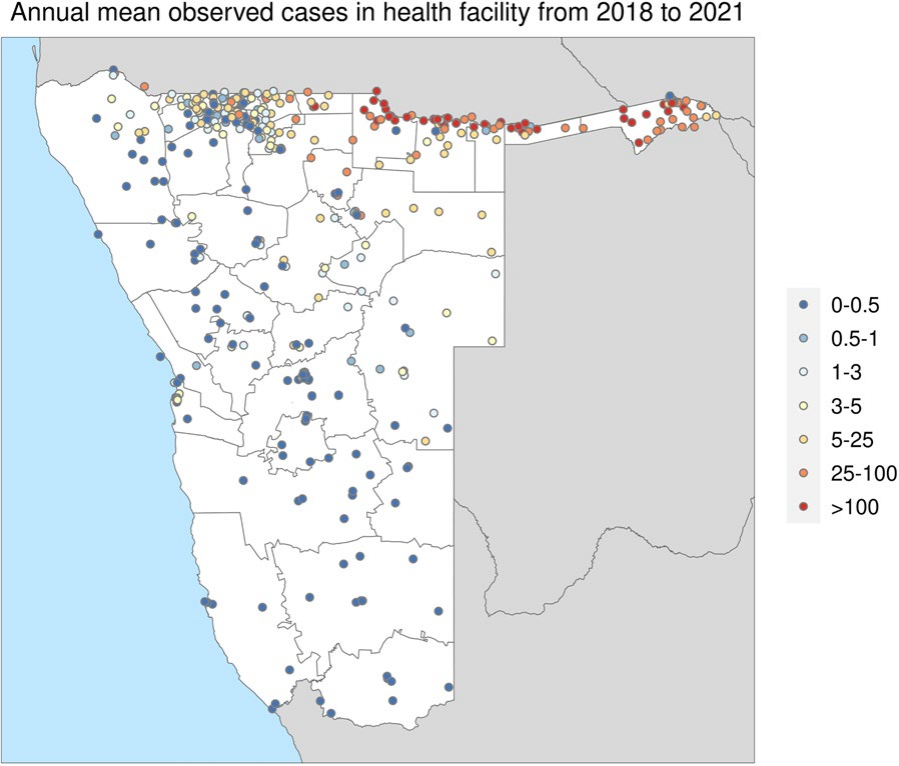
The spatial pattern of malaria endemicity in Namibia based on annual mean observed cases in health facilities from 2018 to 2021

The primary dataset consisted of weekly counts of confirmed local malaria cases of all ages in Namibia, where ‘local’ cases were identified based on the place of residence and an individual’s recent travel history to a malarious region. Patent parasitaemia among febrile care-seekers was detected via rapid diagnostic tests (RDT) or microscopy. The routinely collected case data, in addition to a master facility list, were provided by NVDCP and shared via the Clinton Health Access Initiative (CHAI). A total of 397 geo-located health facilities were matched to reported case data at least once from 2018 to 2021 (Fig. 1). Overall completeness of case reporting among 397 facilities was 81.68% between 2018 and 2021. A number of health facilities in the dataset have the same longitude and latitude, which were assumed to represent hospital in-patient and out-patient clinics or multiple clinics co-located in the same village/town with the same longitude and latitude, and were therefore given unique identifiers and treated as separate entities in the analysis. The period covered by this dataset begins with week 1 in January 2018 and finishes with week 208 in December 2021.

A suite of high-resolution spatial covariates (1 × 1 km) was introduced for statistical modelling and previously described elsewhere [11]. These included accessibility to cities [12], aridity index [13], distance to water (bespoke), elevation [14], enhanced vegetation index (EVI [15]), land cover fraction of savanna [16], day and night land surface temperature (LST [17]), potential evapotranspiration (PET [13]), slope [14], temperature suitability index [18], night-time lights [19], tree cover proportion [20], tasselled-cap brightness (TCB [21]) and tasselled-cap wetness (TCW [21]) (Additional file 1). All products were downloaded from their respective online repositories, gap-filled (where necessary), and aligned to a common grid [22].

Gridded population estimates were sourced from the High Resolution Settlement Layer (HRSL) created by the Connectivity Lab at Facebook (URL: https://www.ciesin.columbia.edu/data/hrsl/). The base resolution for the HSRL is approximately 30 m. Aggregation to 1 km grid-cell was performed by summing values from 30 × 30 m grid squares with centroids lying within the 1 × 1 km pixel boundaries. The population estimates were adjusted to Geo-Referenced Infrastructure and Demographic Data for Development settlement (URL: https://grid3.org/) and then census population data aggregated in different districts in 2020, which were further modified for care-seeking. Consistent with other studies in low transmission settings [23, 24], propensity to seek care was modelled as a three-parameter function of travel-time to nearest health facility. Observations of care-seeking for fever (used as a proxy for care-seeking for malaria) were extracted from the Namibia 2013 Demographic and Health Survey** (**DHS) [25].

An established friction surface was used to create travel-time maps representing time-taken to leave a grid-cell of residence and arrive in the closet cell containing a health facility using the malariaAtlas R package [12, 26]. The parameters of the decay curve were fit using a logistic regression. Note that this care-seeking surface reflects propensity to seek care at *any* facility, with the catchment model explained below determining which facility individual care-seekers are recorded in. Pixels of extremely low population density (< 5 persons per 1 × 1 km grid) were masked and excluded from the analysis to reduce computational cost. These pixels tend to have very little influence in forecasting case counts at facilities in any case: not only is their population share tiny relative to the total catchment population, but they are most prevalent in highly remote areas where distance to facilities is large and care-seeking is low.

### Fine-scale spatial prediction of annual average incidence surface

Inference of incidence rates (which represents local incidence) and seasonality profiles of clinical malaria in Namibia was conducted in two stages. This involved sequentially fitting the spatial and then spatio-temporal dynamics of fluctuations in malaria incidence. Whilst developed foremost to improve computational efficiency and achieve model parsimony, this two-stage method also facilitated the probabilistic separation of epidemic fluctuations from endemic transmission patterns during map making.

Two pre-processing steps were undertaken prior to geospatial statistical modelling. First, Generalized Boosted Regression models based on longitude, latitude and type of health facility were used to impute missing weekly case counts of clinical malaria in health facilities from 2018 to 2021, stratified by age at under and over 5 years old. Second, to avoid multicollinearity, the suite of high-resolution covariates was reduced in dimension via transformation to its first two principal components.

The first stage of modelling approach involved the fitting of a fine-scale, spatial-only geostatistical regression with a flexible catchment sub-model to the total case counts at each facility. The catchment sub-model splits the care-seeking population in a given pixel between multiple neighbouring health facilities in inverse proportion to the square of travel-time distance from pixel to facility and a relative attractiveness weight of each facility; the latter being inferred during model fitting as one free parameter per-facility under a shared regularisation penalty [27]. The catchment sub-model allows patients in any given location attending multiple catchments to split their attendance between multiple closest neighbouring facilities and is used to estimate the catchment population at each facility. The first two principal components from the environmental covariates were introduced as spatially varying coefficients to describe the relationship between covariates and incidence rate. An offset parameter for each health facility was further included in modelling to represent any unexplained variation in the total counts.

The primary output from this model fitting step is an estimate of the annual average incidence rate of malaria cases at pixel level. A secondary output is the annual average case counts at health facility level, which becomes a fixed mean baseline risk surface during the second stage of the analysis (described below). As modelling is done within a Bayesian framework, exceedance surfaces to represent uncertainty were created for pixel outputs illustrating the posterior probability of the incidence rate in each pixel above the threshold of 1 case per 1000 PYO, based on the national strategic plan guideline. Non-exceedance maps present the posterior probability of the incidence rate in each pixel below the threshold of 1 case per 10,000 PYO.

The spatial-only geostatistical regression of the first-stage was implemented with the Template Model Builder (TMB) and Integrated Nested Laplace Approximation (INLA) R packages [28, 29] using a Poisson likelihood and Laplace approximation of the random fields. Namely, ${f}_{\text{slope}}({\text{loc}}_{i})$ which is a bivariate-valued random field providing a pair of multiplicative factors per-pixel against the first two principal components of the covariates, ${X{\prime}}_{\text{PCA}.\text{cov}}$, and ${f}_{\text{intercept}}({\text{loc}}_{i})$ which acts as a spatially varying offset. Additional parameters were the catchment attractiveness weights (${W}_{j}$) and the offset terms of each health facility (${\text{offset}}_{j}$). The posterior approximation of other hyper-parameters is presented as the mean of a series of draws from Multivariate Normal against their densities under this distribution. The structure of this spatial model is described in hierarchical Bayesian notation below.

$${\text{annual cases}}_{j} \sim \text{Poisson} \left(\text{mean}= {\text{expected cases}}_{j}\right)$$

$$\text{e}{{\text{xpected}}_{\text{cases}}}_{j}= \sum_{i}{C}_{i\to j}\times {\text{care}-\text{seeking population}}_{i}\times {I}_{i}\times {\text{offset}}_{j}$$

$${C}_{i\to j} \propto \frac{{W}_{j}}{{T}_{i\to j}^{2}}, \, \, \,\;\; \text{log}{W}_{j} \sim \text{Normal}(0,{ 0.25}^{2})$$

$$\log I_{i} = c + ~X_{{{\text{PCA}}.{\text{cov}}}}^{'} \times ~f_{{{\text{slope}}}} \left( {{\text{loc}}_{i} } \right) + ~f_{{{\text{intercept}}}} \left( {{\text{loc}}_{i} } \right)$$

$${f}_{\text{slope}}\left(.\right) \sim \text{Gaussian Process}\, ({\text{range}}_{\text{slope}}, {\text{scale}}_{\text{slope}})$$

$${f}_{\text{intercept}}\left(.\right) \sim \text{Gaussian Process}\, ({\text{range}}_{\text{int}}, {\text{scale}}_{\text{int}})$$

$${\text{log range}}_{\text{slope}},{\text{log range}}_{\text{int}} \sim \text{Normal}(1, {0.5}^{2})$$

$${\text{log scale}}_{\text{slope}},{\text{log scale}}_{\text{int}} \sim \text{Normal}\left(-1, {0.5}^{2}\right)$$

$${\text{log offset}}_{j}\sim \text{Normal}\left(0,{ 0.5}^{2}\right), c \sim \text{Improper Uniform}$$

### Spatio-temporal modelling of seasonal fluctuations in incidence

For the second stage of modelling, the average case counts were fixed at each health facility estimated in the previous step and fit a facility-level spatio-temporal geostatistical model to explain the temporal variation of weekly incidence rates from 2018 to 2021. A Generalized Additive model was introduced below with an independent and identically distributed (iid) random effect for each health facility (${f}_{1}$), a tensor product smooth of the coordinates of health facility and week (${f}_{2}$), with baseline weekly risk surface for each health facility from the first stage as an intercept (${\text{expected cases}}_{j}$).

$${\text{log case}}_{jk} \sim \text{log}{(\text{expected cases}}_{j})+{f}_{1}({\text{fac}\; \text{ID}}_{j})+{f}_{2}\left({\text{fac coordinates}}_{j}, {\text{week}}_{k}\right)$$

${fac\; ID}_{j}$ is the number of facilities, ${week}_{k}$ is the number of weeks from 2018 to 2021, ${f}_{1}(. ) \sim {\text{iid}}$ Normal random effect, ${f}_{2} (.) \sim$tensor product smooth using thin plate regression splines for coordinates and cubic regression splines for week.

The mean predicted incidence rates at health facility level from the first stage were taken as baseline exposure, and the spatio-temporal model above was fitted to estimate weekly incidence in each facility. To assess the predictive performance of this second modelling process, model validation was performed by holding out random 12.5% of time points (26 out of 208 weeks) for each facility. The model was fitted on the remaining 87.5% of the data (182 out of 208 weeks for each facility) and used to predict the held-out set. In addition, in the first stage modelling, we obtained the lower quantile (2.5%), mean and upper quantile (97.5%) of expected incidence surfaces at health facilities from a series of draws of Multivariate Normal distribution against their densities. After fixing them as different intercept parameters at each health facility during the second stage modelling, a sensitivity analysis was conducted to compare the distribution patterns of predicted cases and annual incidence rates at health facilities between three spatio-temporal models.

The weekly mean expected incidence was aggregated from health facility level to district level. This analysis used 35 Namibian health districts for aggregation purposes, in order to align with the operational requirements of the NVDCP. Populations may cross district boundaries to attend health facilities outside their usual district of residence. The catchment model accounts for this flux of cases by explicitly modelling incidence in the community, with no boundary effects applied to prevent catchments crossing administrative boundaries [30].

## Results

### Fine-scale spatial endemicity surface

**Fig. 2:**
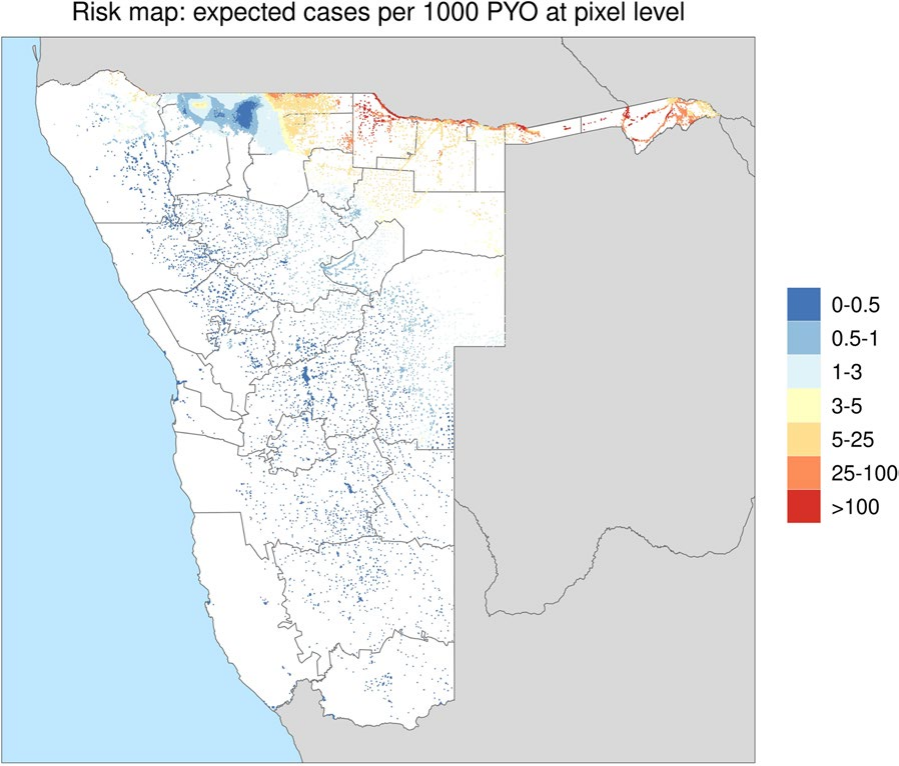
Posterior (geometric) mean of the incidence rate of malaria in Namibia at 1 × 1 km resolution, presented as expected cases per 1000 PYO generated in the first stage of spatial modelling

Overall, there were 6797 and 45,535 locally reported malaria cases under and over 5 years old, respectively, between 2018 and 2021 in Namibia. A total of 342 missing weekly case counts under 5 years and 1751 missing weekly case counts over 5 years in health facilities were imputed separately and used to fill the reporting gaps. The case counts for all ages were combined and modelled in the first stage. When the suite of covariates was converted to principal components during the pre-modelling step, the covariates that gave the most weights to the first principal component were aridity index, EVI and LST Day, whereas the covariates with the most weights to the second principal component were TCB, TCW and LST Night. The first two principal components of all covariates at pixel level were fitted in the model as linear predictors having slopes that varied spatially and randomly. Posterior geometric mean estimate of clinical incidence rate in the number of cases per 1000 PYO was shown in Fig. 2.

Posterior probabilistic maps of exceedance (Additional file 2A) and non-exceedance (Additional file 2B) of the spatial variation in risk (as estimated in the first stage modelling) were provided to the NVDCP to assist in interpretation of the risk maps. The results revealed a high degree of heterogeneity in malaria burden in Namibia: the southern part of the country was essentially malaria free, but there remained a number of high burden communities in the northern part, primarily the Zambezi region between Angola and Zambia.

### Spatial pattern at district level and seasonality profile

The overall predicted incidence was 11.23 (95% CI 8.88–13.58), 1.25 (95% CI 0.63–1.88), 5.42 (95% CI 3.80–7.04) and 5.30 (95% CI 4.04–6.56) per 1000 PYO in 2018, 2019, 2020 and 2021, respectively. There was a high correlation between the observed and predicted weekly case counts in the held-out set at health facilities with the Pearson’s coefficient of 0.907 (95% CI 0.904–0.911) (Additional file 3A), indicating good predictive out-of-sample performance. Based on the lower quantile (2.5%), mean and upper quantile (97.5%) of predicted incidence surfaces at health facilities in the first stage modelling, the sensitivity analysis of the second stage modelling showed almost identical distribution of expected cases (Additional file 3B) and predicted annual incidence rates, suggesting its insensitivity to model intercept parameters.

**Fig. 3:**
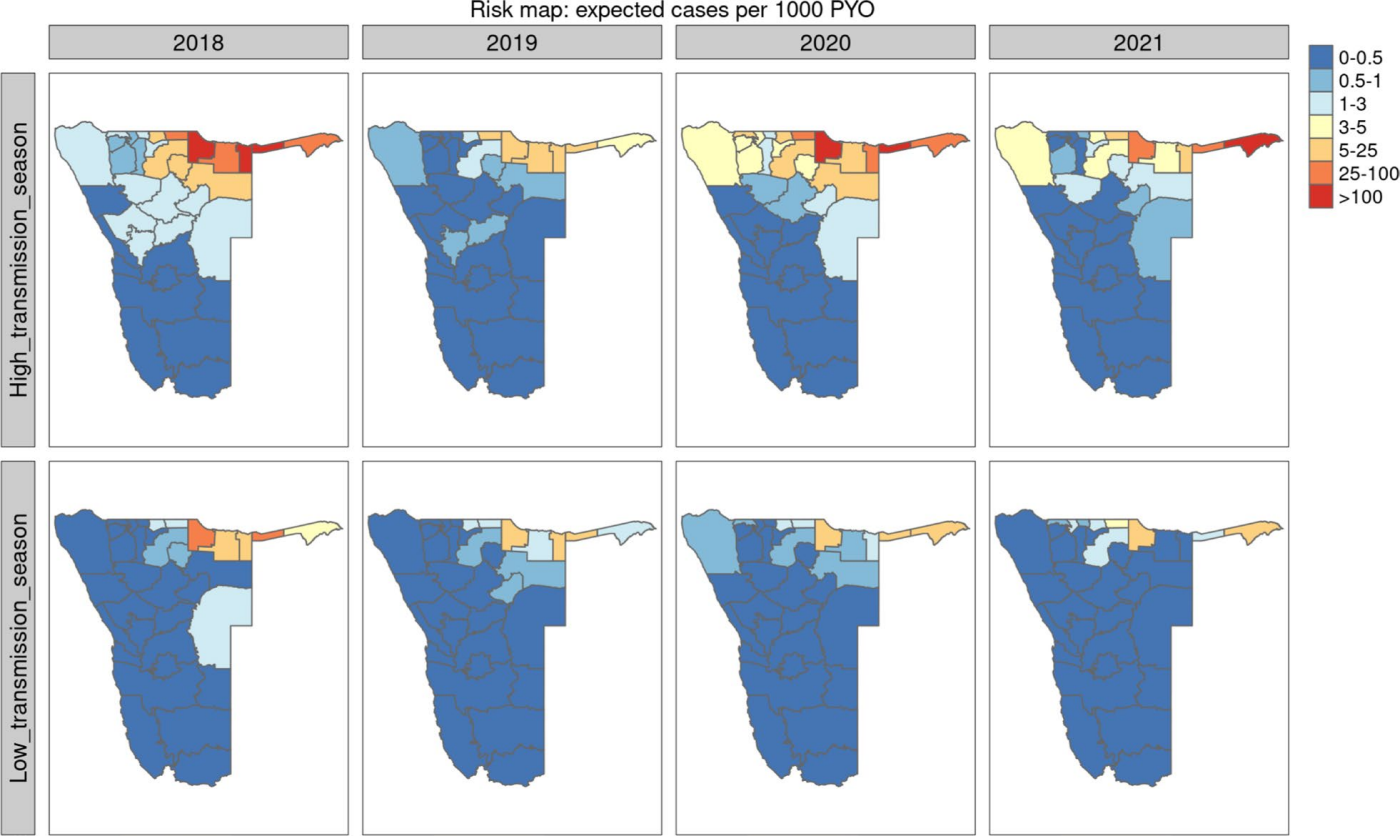
The incidence rate of malaria in Namibia aggregated to district in high transmission season (week 1–week 26, top) and low transmission season (week 27–week 52, bottom) from 2018 to 2021, as estimated in the second stage of spatio-temporal modelling at health facility level

In risk maps of average expected cases per 1000 PYO from 2018 to 2021, the mean incidence rates were high (10.66; 95% CI 8.24–13.08) in the first half year from January to June defined as high transmission season, but low (0.94; 95% CI 0.43–1.45) in the second half year from July to December defined as low transmission season (Fig. 3). A seasonal profile was inferred across most districts of the country, with cases rising from early January (or a few weeks in the previous year) to a peak around early April (~ week 14) and then declining rapidly to a low level from July to the end of the year from 2018 to 2021 in Nankudu, Katima Mulilo, Andara, Nyangana, Rundu, Okongo, Eenhana and Omuthiya (Additional file 4). Annual transmission patterns oscillated between a high transmission season (the first six months of the calendar year) and low transmission season (the last six months of the calendar year), showing a clear seasonal pattern. After aggregation to health districts, 2.4% of predicted cases were estimated to seek care at health facilities outside of their district of residence—these were included in the relevant health facility totals for each district, but were otherwise included in their district of estimated residence for community metric calculations. At health-district aggregate, root mean squared error was 0.0024 for the first stage modelling and 0.029 after final spatio-temporal model fit.

**Fig. 4:**
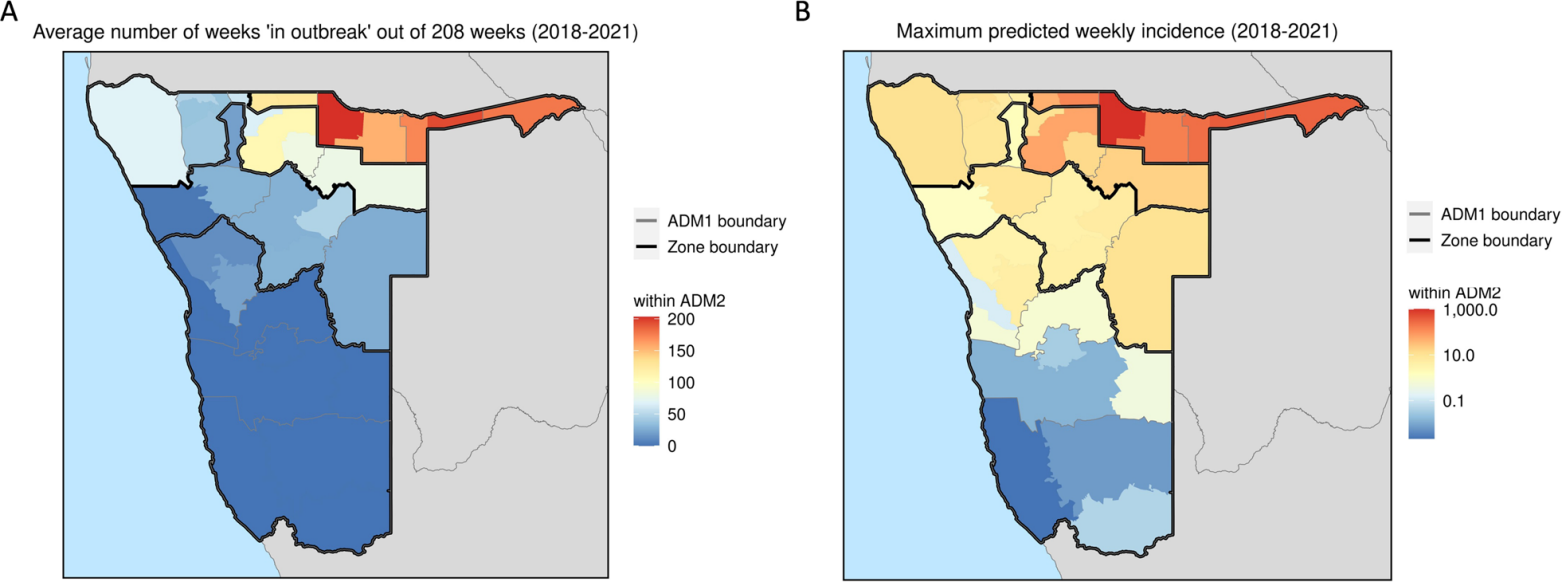
Two maps of incidence rates of malaria in Namibia aggregated to district (ADM2), which is further illustrated within the boundary of regions (ADM1 boundary: grey) and four defined transmission zones (Zone boundary: black). **A** The average number of weeks in ‘outbreak’ conditions over the study period, where an ‘outbreak’ is defined to be a week where incidence rate exceeded 1 case per 1000 PYO. **B** maximum predicted weekly incidence indicated as cases per 1000 PYO from 2018 to 2021

There was a high degree of spatial heterogeneity in malaria incidence in Namibia, with incidence far higher in the north of the country. To assist interpretation of risk maps, we defined four malaria transmission zones aggregated from districts and regions and categorized from north to south with moderate (Zone 1), low (Zone 2), very low (Zone 3) and non-receptive (Zone 4) risks according to predicted incidence thresholds and geographic locations based on Namibian current strategic plan (Fig. 4). The mean predicted incidence was over 5 per 1000 population in Zone 1 districts, between 1 and 5 in Zone 2 districts, between 0 and 1 in Zone 3 districts and 0 in Zone 4 districts.

Figure 4 illustrates the utility of these estimates for understanding inter- and intra- annual variation in hotspot emergence. Figure 4A summarises the time each district spent in ‘outbreak’ conditions over the study period, where an 'outbreak' is defined to be a week where incidence rate exceeded 1 case per 1000 PYO. For example, Andara was classified to be 93.75% ‘in outbreak’ over the whole study period. Figure 4B, meanwhile, displays maximum predicted weekly incidence in each district from 2018 to 2021. Whilst regular outbreaks are experienced in Nankudu, occasional large spikes in incidence were predicted further south, including a peak of 10.46 cases per 1000 PYO of Gobabis in October 2018.

**Fig. 5:**
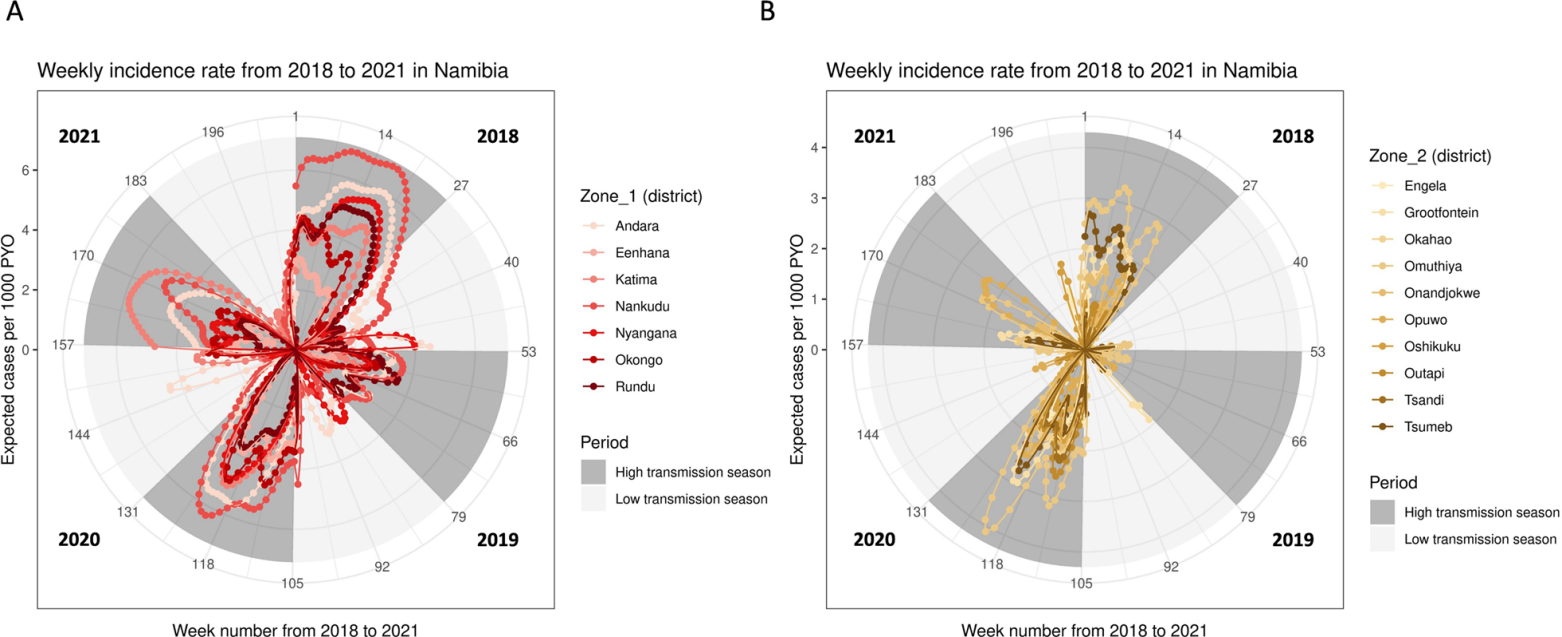
Weekly predicted incidence rates from 2018 to 2021 in Namibia, aggregated to district in **A** the moderate transmission Zone 1 and **B** the low transmission Zone 2 in circular plots overlaid with high and low transmission seasons

Weekly predicted incidence is presented in districts in four transmission zones (Additional file 5). Both moderate transmission Zone 1 (Fig. 5A) and low transmission Zone 2 (Fig. 5B) displayed marked seasonal profiles, with the exception of 2019 where very low levels of transmission persisted throughout the year. Some local epidemic occurrence of malaria in specific districts in the low transmission season is observed. The relationship between observed, imputed and predicted cases per 1000 PYO were illustrated to indicate a good model fit (Additional files 6 and 7). A heatmap of the percentage of population living in districts with observed and predicted cases over 5 per 1000 PYO is illustrated weekly from 2018 to 2021 (Additional file 8), highlighting substantial interannual variation in distribution of risk.

## Discussion

Namibia has made remarkable progress in its efforts to eliminate malaria with the target to eliminate malaria in the northern regions by 2030, and a current strategic plan aiming for zero local cases by 2027. The objective of this study was to provide a comprehensive analysis of spatio-temporal fluctuations in malaria incidence at a fine spatial and temporal scale. To achieve this, a modelling approach was employed combining spatially varying environmental covariates, estimates of care-seeking behaviour, and a catchment sub-model to infer community incidence from facility-based data [24, 31, 32].

Malaria clinical cases from passive surveillance data at heath facilities for disease risk mapping are usually incomplete and under-reported [33]. To mitigate this source of bias, missing weekly case counts were imputed using a Generalized Boosted Regression model during the pre-processing step for the subsequent geostatistical modelling. The first stage of the modelling process generated a high-resolution (1 km grid-cell) map of spatial variation of malaria incidence in Namibia. The granularity of this map is suitable for informing the targeting of precision interventions to transmission hotspots. In the second stage, the framework was expanded to incorporate spatio-temporal weekly variations in risk spanning from 2018 to 2021 in every health facility. The maximal temporal resolution of this approach (weekly) provides valuable support for outbreak detection of anomalous districts, reactive case identification and hotspot prevention.

While previous geo-statistical and risk factor studies mostly focused on northern Namibia [23, 24, 34–38], the malaria risk mapping developed in this study identifies the hotspots of malaria transmission in the whole country, including most districts in the north-eastern Namibia defined as moderate transmission Zone 1 and some districts in central Namibia defined as a low transmission Zone 2. A full national based approach allows for a more accurate estimations of catchment populations. Large swathes of Namibia are sparsely settled, meaning catchments are forced to cover wider areas. The predicted annual incidence in Namibia in 2018, 2019, 2020 and 2021 was higher than previously estimated incidence in the northern regions from January 2012 to May 2014 (less than 1 case per 1000 PYO) [24], reflecting increased focal outbreaks in the three north-eastern-most regions in recent years. Some regions with extremely high incidence rates might be due to highly mobile populations where large numbers of cases came from non-residents, e.g. travellers, seasonal migrants and agricultural workers, who may not have been accurately captured in the gridded population surfaces used in this analysis. A higher risk of malaria has been observed in cross-border travellers in northern Namibia, compared with those non-travellers or domestic travellers [34, 39, 40]. Potentially, integrating mobile phone record data and malaria risk mapping would provide the possibility to measure internal and overseas migration patterns and adjust for the estimates of population numbers at multiple spatial and temporal scales [39, 41, 42]. Concerted effects with neighbouring countries are essential to put in place to realize the local elimination targets by 2027.

The predicted weekly incidence estimated the highest incidence between January and June in districts of Nankudu, Katima Mulilo, Andara, Nyangana, Rundu, Okongo, Eenhana and Omuthiya bordering Angola, Zambia and Botswana, with lower values estimated in the second half of year. The seasonal pattern of incidence was previously shown to be associated with many environmental risk factors including lower temperature, higher rainfall and increased vegetation [34]. For example, the seasonal profiles in the moderate and low transmission zones starting from December with seasonal peaks persisting up to April coincide with the rainy period in Namibia [24, 43, 44]. The drop in incidence in 2019 may also be a response to an unusually dry year [45]. Delays in vector control interventions in northern Namibia likely impacted burden during the COVID-19 pandemic in 2020 and 2021 [46].

As malaria transmission is heterogenous across Namibia, four transmission zones of moderate, low, very low and non-receptive risks, were defined aiming to support the Namibian NVDCP for specific operational and planning purposes. High resolution monthly maps of malaria transmission intensity have been previously used for planning, monitoring, evaluation, and resource allocation in northern Namibia [24]. These high-resolution spatio-temporal profiles of malaria endemicity can be used to estimate the disease burden locally and assess the effectiveness of interventions and progress towards its elimination nationally and sub-nationally.

The two-stage spatio-temporal modeling approach demonstrated in this research provides a robust framework for malaria risk mapping that can be feasibly adapted to other countries. Ensuring the regular and accurate weekly capture of case data is paramount. This can be facilitated through strengthening existing health information systems, ensuring rigorous data collection protocols, and leveraging technology for real-time data entry and validation. Infrastructure such as geo-located health facilities and an updated master facility list are critical. In addition, early collaboration with local health authorities and stakeholders is essential to tailor the model to the specific epidemiological and geographical context of the new setting and more importantly to understand the quality of the case data from their national surveillance system. By addressing these baseline data and infrastructure needs, the two-stage approach can provide actionable insights for targeted intervention and support malaria elimination efforts in diverse contexts.

This study has several limitations. Firstly, an aggregate of diagnostic methods in this study makes it difficult to ascertain the proportion of RDT or microscopy and thus unable to adjust for diagnostic differences for patent parasitaemia. There is significant uncertainty around the population denominator—Namibia has a highly mobile population, and low treatment-seeking behaviours. The imputation method used during the pre-processing step may not capture the true patterns of case missingness, and thus may introduce bias in the subsequent modelling. Due to computational limitations, spatial surfaces were estimated for annual average case counts between 2018 and 2021 at both pixel and health facility levels in the first stage modelling. Temporal variation was modelled at health facility level and aggregated to health districts rather than at fine scale pixel level, though we note that operational decisions on targeting interventions will be largely made at this health district level by the NVDCP.

## Conclusions

This novel two-stage spatio-temporal approach provides valuable support for a local weekly detection tool for hotspot identification and monitoring in malaria risk patterns and tailored intervention strategies in Namibia. With the advent of advances in geostatistical techniques and computational efficiency, approaches in malaria risk mapping at increasingly high resolution continue to be developed and used to inform public health policy.

## Supplementary Information

Additional file 1. The selection of covariates in the study

Additional file 2. Exceedance and non-exceedance maps of malaria incidence in Namibia. **A** The posterior probability the incidence rate exceeds 1 case per 1000 PYO in each pixel under the first stage spatial model. **B** The posterior probability the incidence rate does not exceed 1 case per 10,000 PYO in each pixel under the first stage spatial model.

Additional file 3. Validation and sensitivity analysis of the second stage spatio-temporal modelling process at health facility level. **A** A Generalized Additive Model is fitted on random 87.5% of the weekly incidence count data in each facility from 2018 to 2021, which is then used to predict the held-out set containing 12.5% of the data. The scatter plot illustrates the correlation between observed cases and predicted cases in each facility in the held-out set. A linear regression line is indicated in red. **B** Histograms of the number of expected cases in the second stage modelling based on the lower quantile, mean and upper quantileof predicted incidence surfaces at health facilities from the first stage modelling.

Additional file 4. Maps of incidence rates of malaria in Namibia aggregated to district in the first and second half yearfrom 2018 to 2021, obtained through the second stage of spatio-temporal modelling at health facility level

Additional file 5. Weekly predicted incidence rates of malaria from 2018 to 2021 in Namibia aggregated to district in moderate, low, very low and non-receptive transmission zones

Additional file 6. Weekly observed, imputed and predicted cases per 1000 PYO in districts from 2018 to 2021

Additional file 7. The relationship between total observed and predicted cases per 1000 PYO in districts between 2018 and 2021

Additional file 8. Heatmaps of **A** observed and **B** predicted rates showing the weekly percentage of population living in districts with over 5 cases per 1000 PYO from 2018 to 2021.

## Data Availability

No datasets were generated or analysed during the current study.

## Abbreviations

PYO: Person-Years-Observed
NVDCP: National Vector-Borne Diseases Control Programme
RDT: Rapid Diagnostic Test (RDT)
CHAI: Clinton Health Access Initiative
EVI: Enhanced Vegetation Index
LST: Land Surface Temperature
PET: Potential Evapotranspiration
TCB: Tasselled-Cap Brightness
TCW: Tasselled-Cap Wetness
HRSL: High Resolution Settlement Layer
DHS: Demographic and Health Survey
TMB: Template Model Builder
INLA: Integrated Nested Laplace Approximation
