# Supplementary material for: High-resolution spatio-temporal risk mapping for malaria in Namibia: a comprehensive analysis
Source: Malar J. 2024 Oct 5;23:297. doi: 10.1186/s12936-024-05103-w (PMC11452985; doi:10.1186/s12936-024-05103-w)
Supplement: Supplementary file 1 — Additional file 1. The selection of covariates in the study [file 12936_2024_5103_MOESM1_ESM.pdf]

Additional file 1.

Our modelling approaches use a range of environmental and socio-demographic covariates with a spatial resolution of 1 km × 1 km, known to influence and impact malaria outcomes. The selection of these covariates was informed by a comprehensive review that identified robust associations with malaria (1). The details of the covariates are described in Table S1. Dynamical covariates were based on the average year between 2018–2021.

In the first stage of modelling, we used the first two principal components of these covariates at pixel level to effectively address multicollinearity by transforming the original correlated covariates into a new set of uncorrelated components.

Table S1. List of covariates used in principal component analysis.

**Covariate Name**	**Description**
Static Covariates	
Elevation	Elevation as measured by the shuttle radar topography mission (SRTM)
Slope	GIS-derived surface calculated from SRTM elevation surface
PET	Potential evapotranspiration
Access to cities	Travel time distance to cities with population > 50,000
AI	Aridity Index
Distance to water	GIS-derived surface that measures distance to permanent and semi-permanent water based on presence of lakes, wetlands, rivers and streams, and accounting for slope and precipitation
Night-time lights	Index that measures the presence of lights from towns, cities and other sites with persistent lighting
TSI	Temperature suitability index for P. falciparum
Land cover fraction of savanna	IGBP-defined fraction coverage of land cover class savanna type
Tree cover proportion	Percentage of forest cover
Dynamic Covariates	
EVI	Enhanced vegetation index
LST Day	Daytime land surface temperature
LST Night	Night-time land surface temperature
TCB	Tasselled cap brightness; measure of land reflectance
TCW	Tasselled cap wetness; measure of land moisture

1. Weiss DJ, Mappin B, Dalrymple U, Bhatt S, Cameron E, Hay SI, et al. Re-examining environmental correlates of Plasmodium falciparum malaria endemicity: a data-intensive variable selection approach. Malaria Journal. 2015;14(1):68.
