# Supplementary material for: High-resolution spatio-temporal risk mapping for malaria in Namibia: a comprehensive analysis
Source: Malar J. 2024 Oct 5;23:297. doi: 10.1186/s12936-024-05103-w (PMC11452985; doi:10.1186/s12936-024-05103-w)

Additional file 2

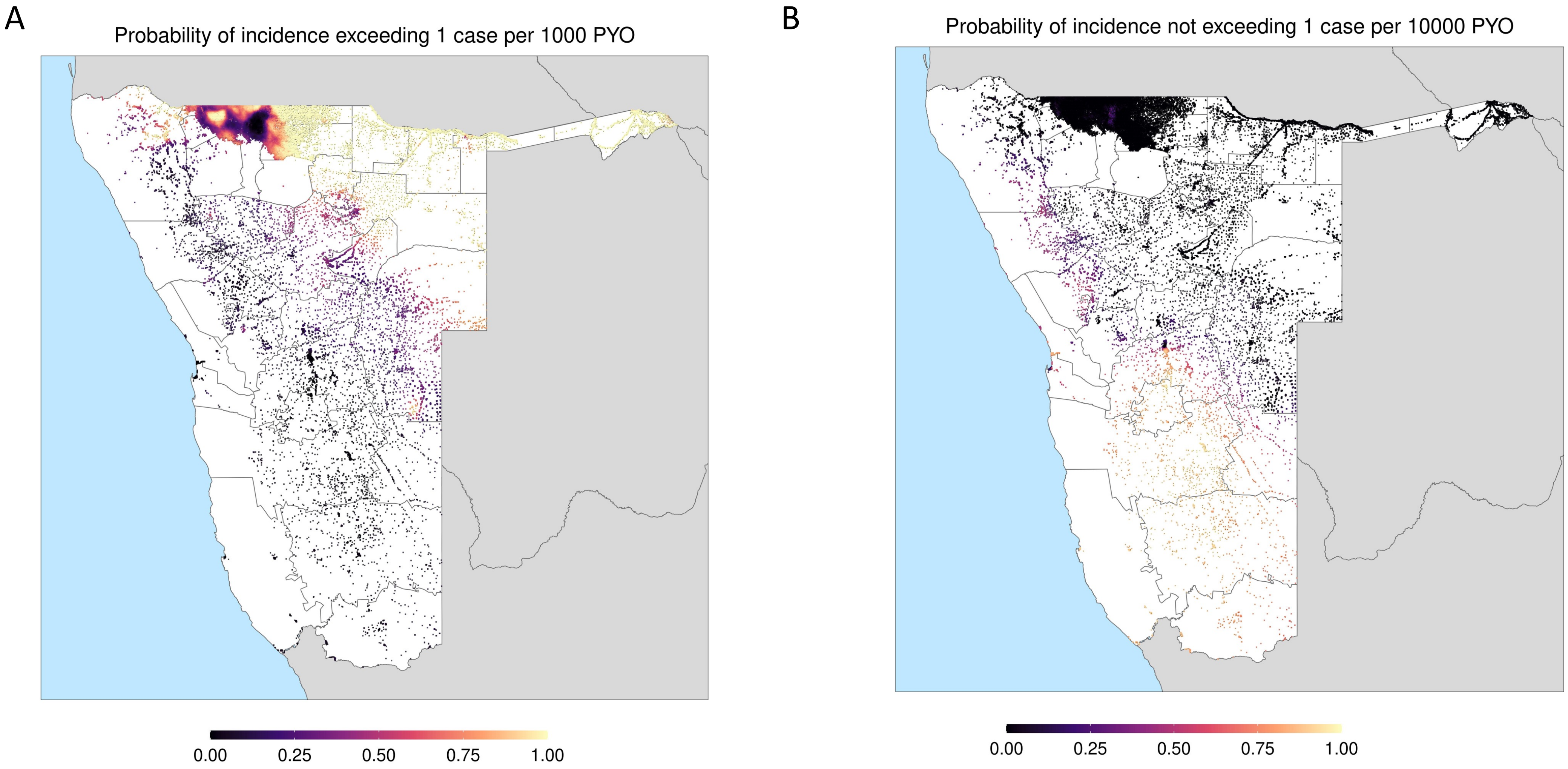

Supplement: Supplementary file 2 — Additional file 2. Exceedance and non-exceedance maps of malaria incidence in Namibia. A The posterior probability the incidence rate exceeds 1 case per 1000 PYO in each pixel under the first stage spatial model. B The posterior probability the incidence rate does not exceed 1 case per 10,000 PYO in each pixel under the first stage spatial model. [file 12936_2024_5103_MOESM2_ESM.pdf]
