# Supplementary material for: High-resolution spatio-temporal risk mapping for malaria in Namibia: a comprehensive analysis
Source: Malar J. 2024 Oct 5;23:297. doi: 10.1186/s12936-024-05103-w (PMC11452985; doi:10.1186/s12936-024-05103-w)

Additional file 3

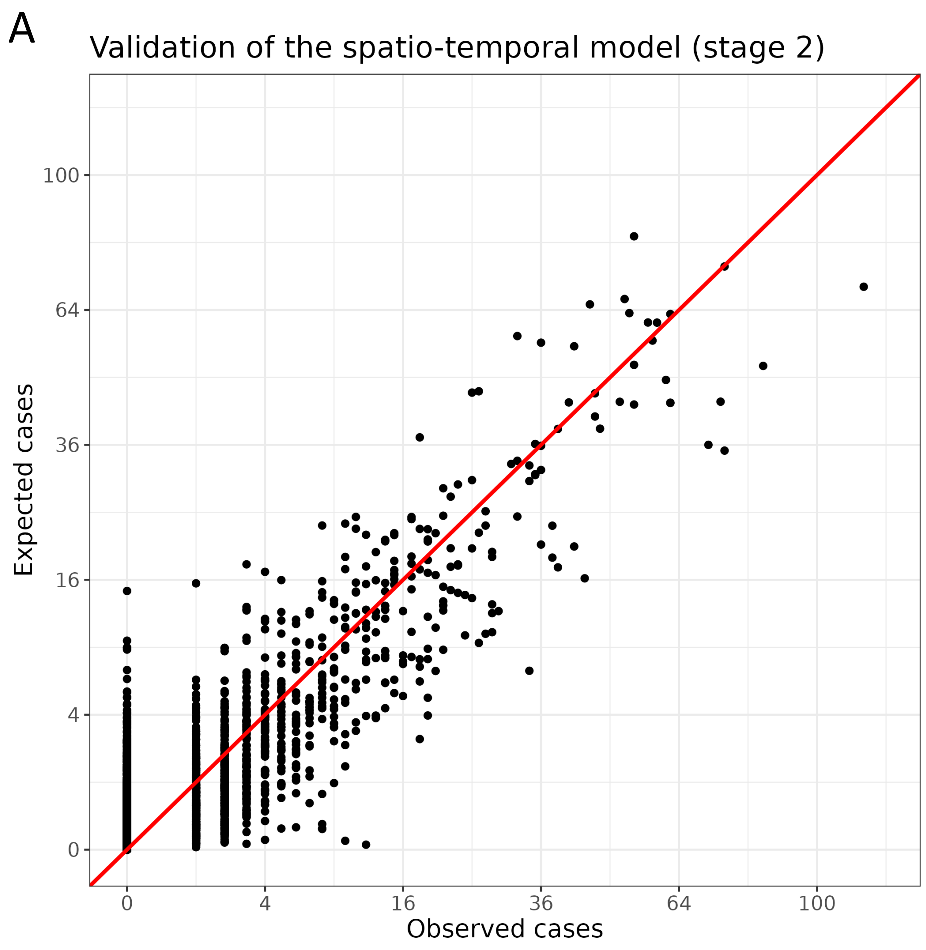

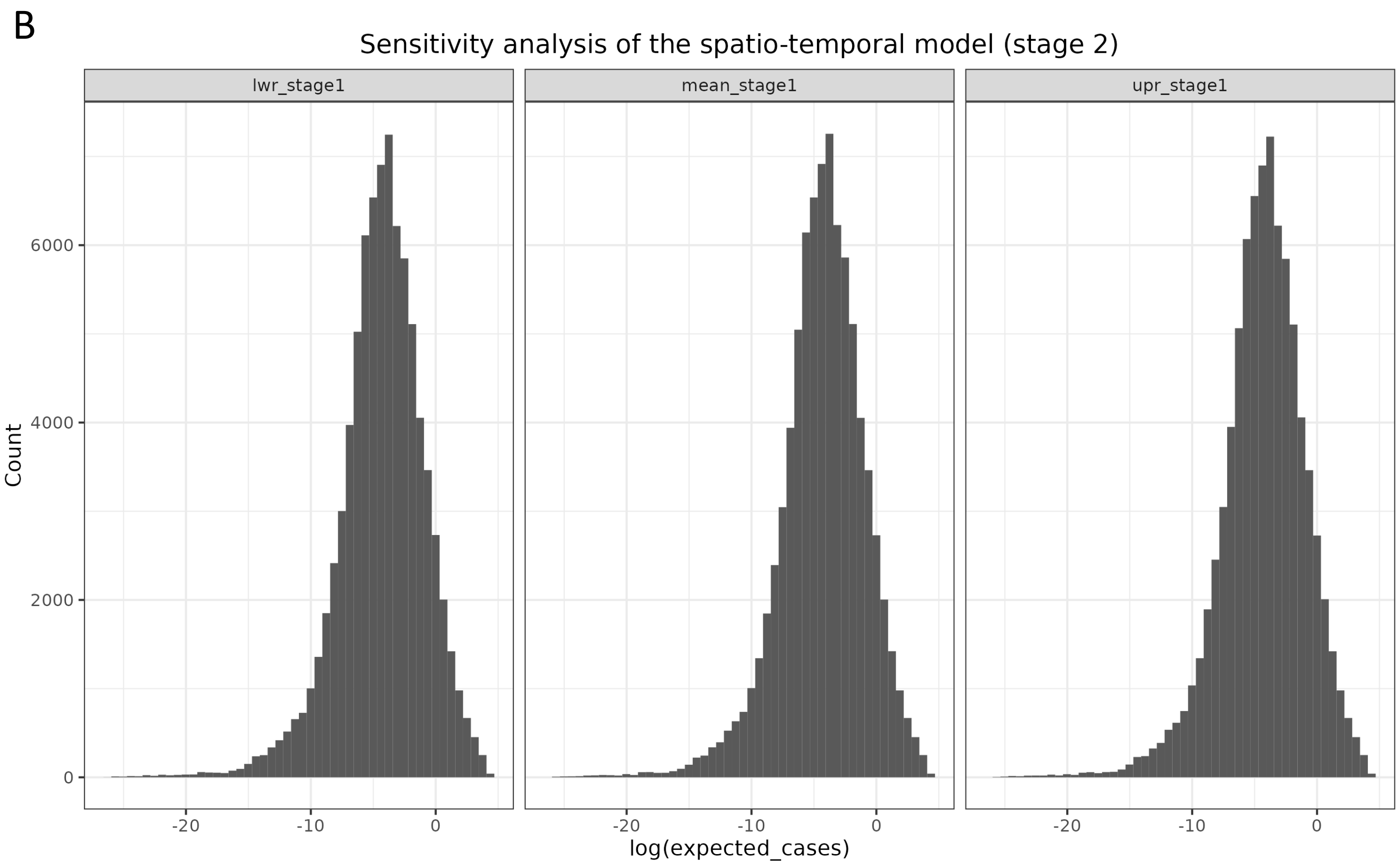

Supplement: Supplementary file 3 — Additional file 3. Validation and sensitivity analysis of the second stage spatio-temporal modelling process at health facility level. A A Generalized Additive Model is fitted on random 87.5% of the weekly incidence count data in each facility from 2018 to 2021, which is then used to predict the held-out set containing 12.5% of the data. The scatter plot illustrates the correlation between observed cases and predicted cases in each facility in the held-out set. A linear regression line is indicated in red. B Histograms of the number of expected cases in the second stage modelling based on the lower quantile, mean and upper quantileof predicted incidence surfaces at health facilities from the first stage modelling. [file 12936_2024_5103_MOESM3_ESM.pdf]
