# Supplementary figures and images for: High-resolution spatio-temporal risk mapping for malaria in Namibia: a comprehensive analysis
Source: Malar J. 2024 Oct 5;23:297. doi: 10.1186/s12936-024-05103-w (PMC11452985; doi:10.1186/s12936-024-05103-w)

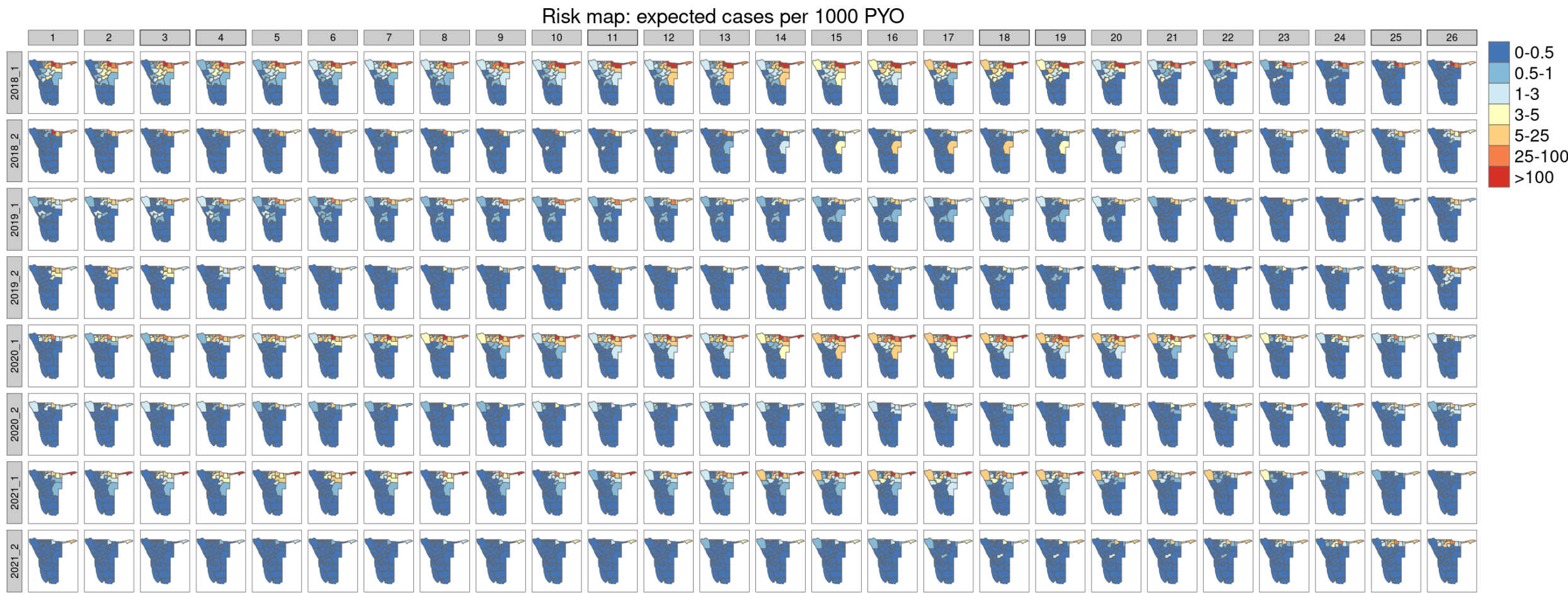

Supplement: Supplementary file 4 — Additional file 4. Maps of incidence rates of malaria in Namibia aggregated to district in the first and second half yearfrom 2018 to 2021, obtained through the second stage of spatio-temporal modelling at health facility level [file 12936_2024_5103_MOESM4_ESM.pdf]

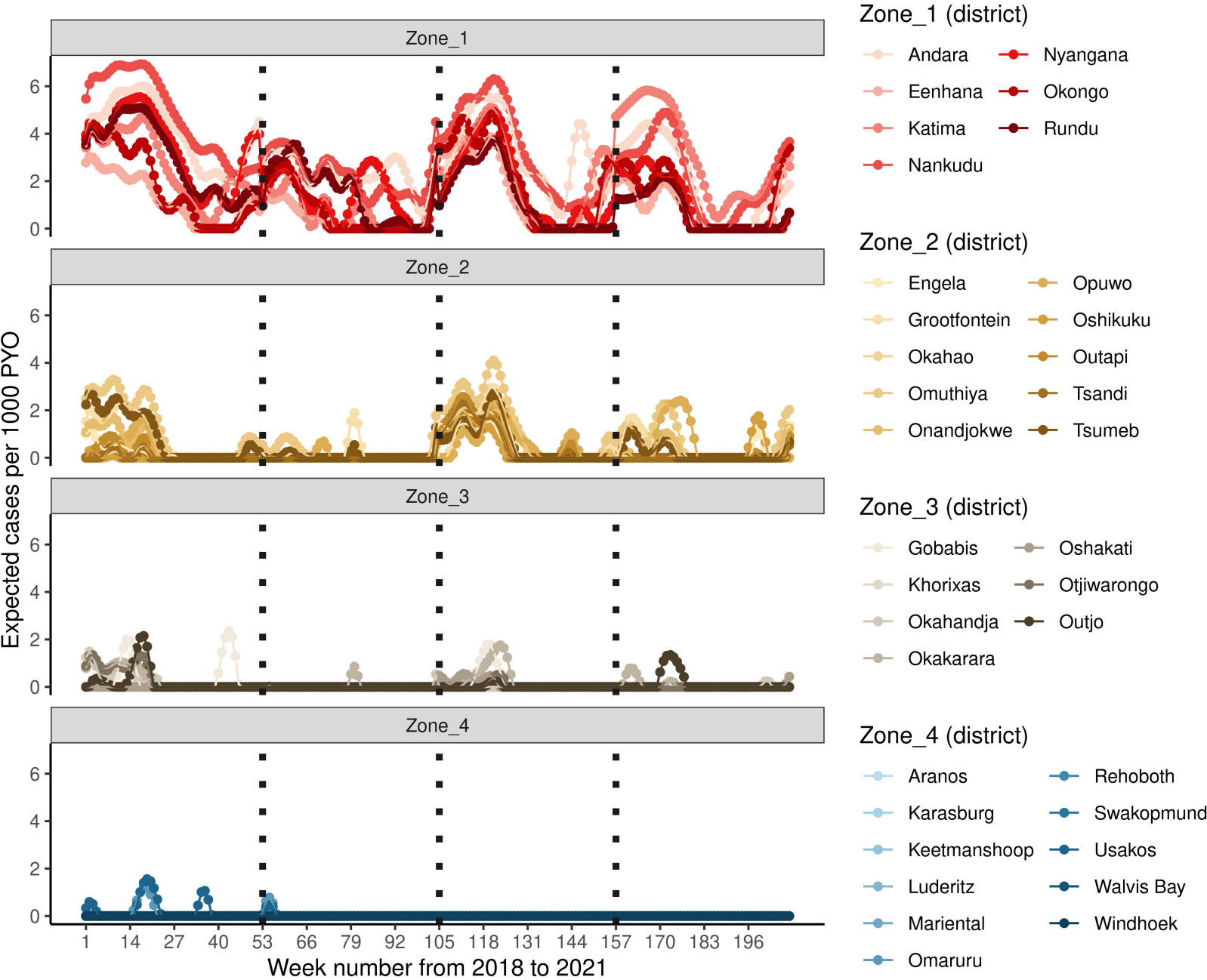

Supplement: Supplementary file 5 — Additional file 5. Weekly predicted incidence rates of malaria from 2018 to 2021 in Namibia aggregated to district in moderate, low, very low and non-receptive transmission zones [file 12936_2024_5103_MOESM5_ESM.pdf]

Additional file 6

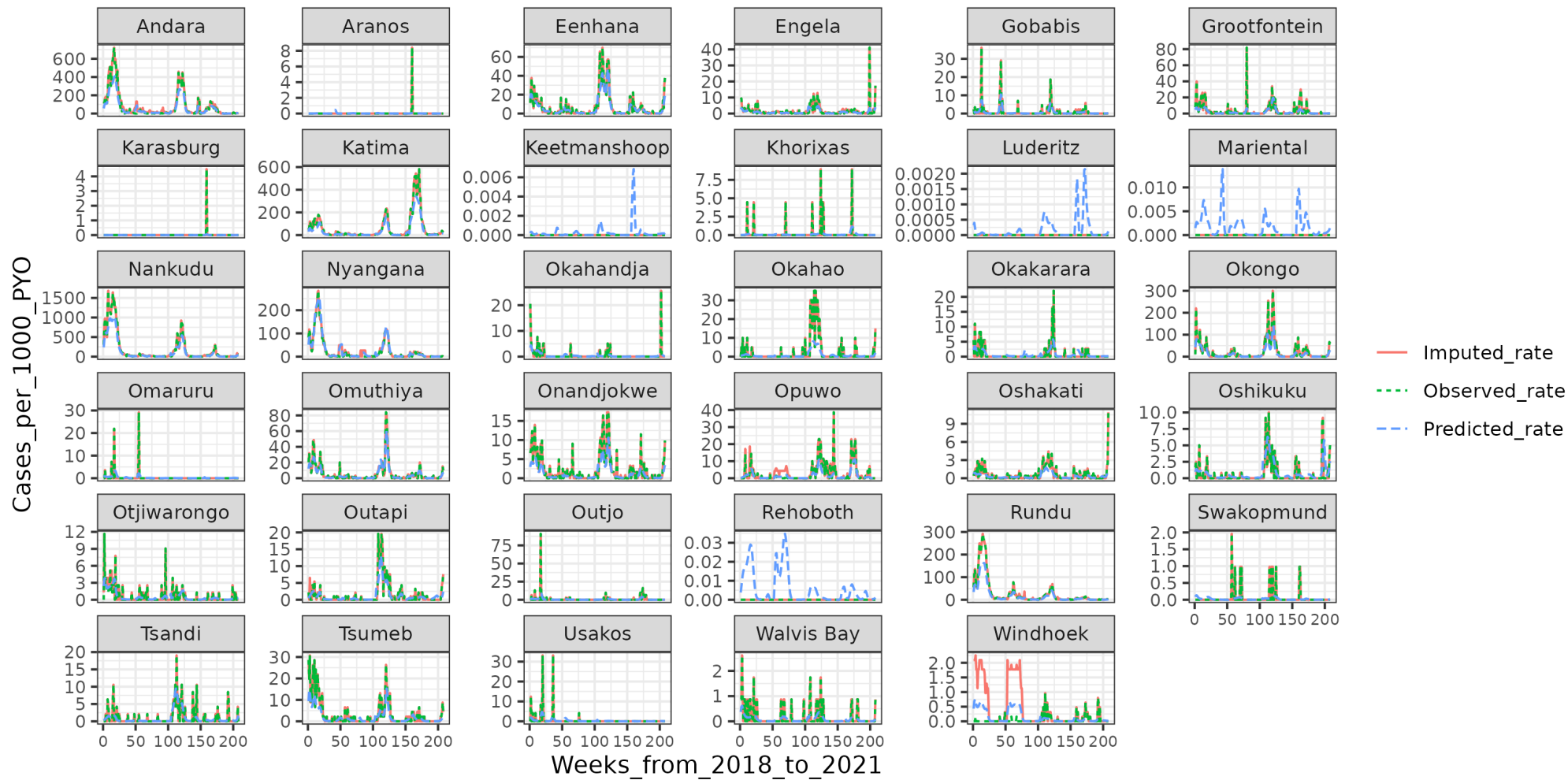

Supplement: Supplementary file 6 — Additional file 6. Weekly observed, imputed and predicted cases per 1000 PYO in districts from 2018 to 2021 [file 12936_2024_5103_MOESM6_ESM.pdf]

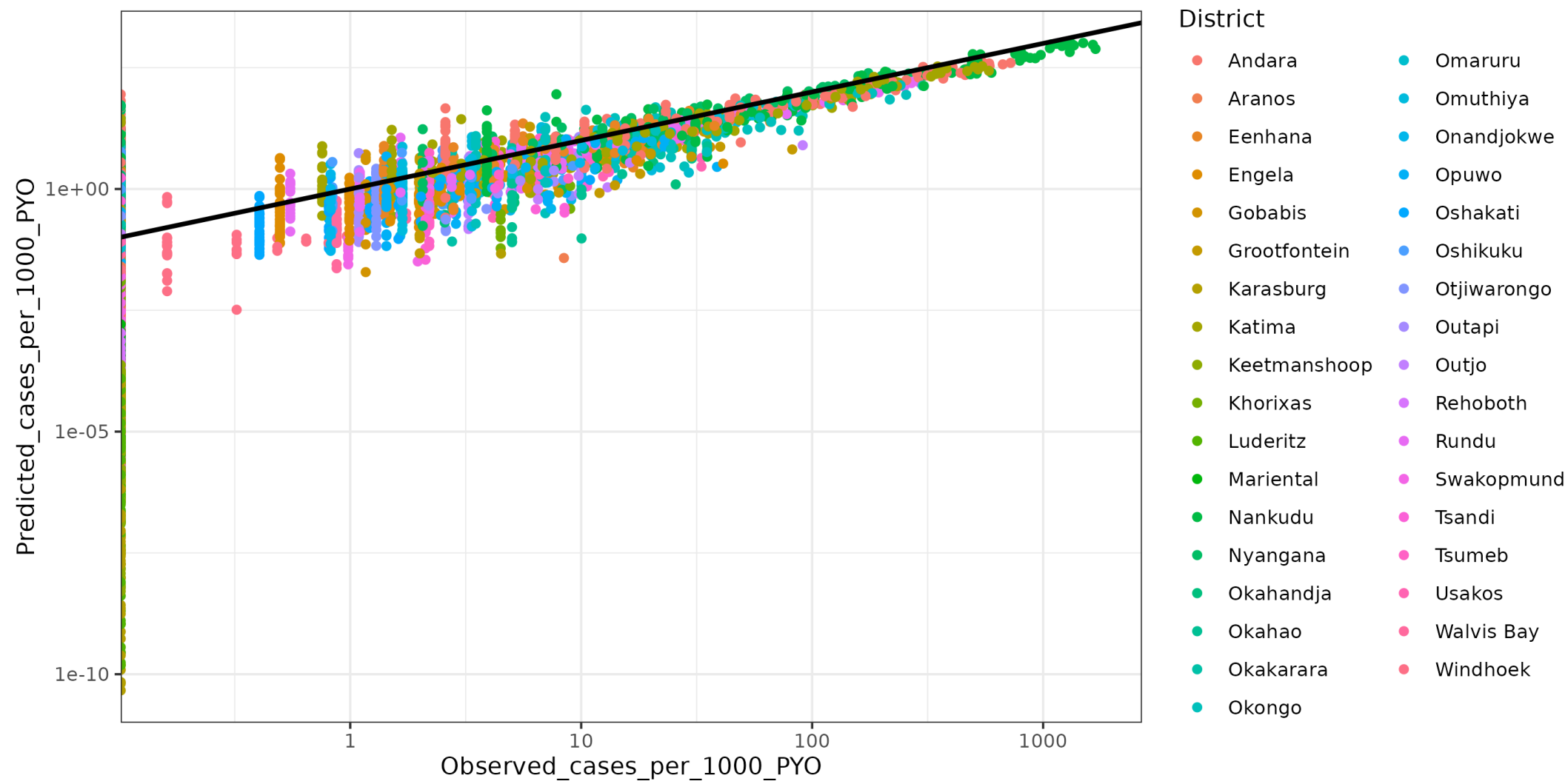

Supplement: Supplementary file 7 — Additional file 7. The relationship between total observed and predicted cases per 1000 PYO in districts between 2018 and 2021 [file 12936_2024_5103_MOESM7_ESM.pdf]
