# Supplementary material for: High-resolution spatio-temporal risk mapping for malaria in Namibia: a comprehensive analysis
Source: Malar J. 2024 Oct 5;23:297. doi: 10.1186/s12936-024-05103-w (PMC11452985; doi:10.1186/s12936-024-05103-w)

Additional file 8

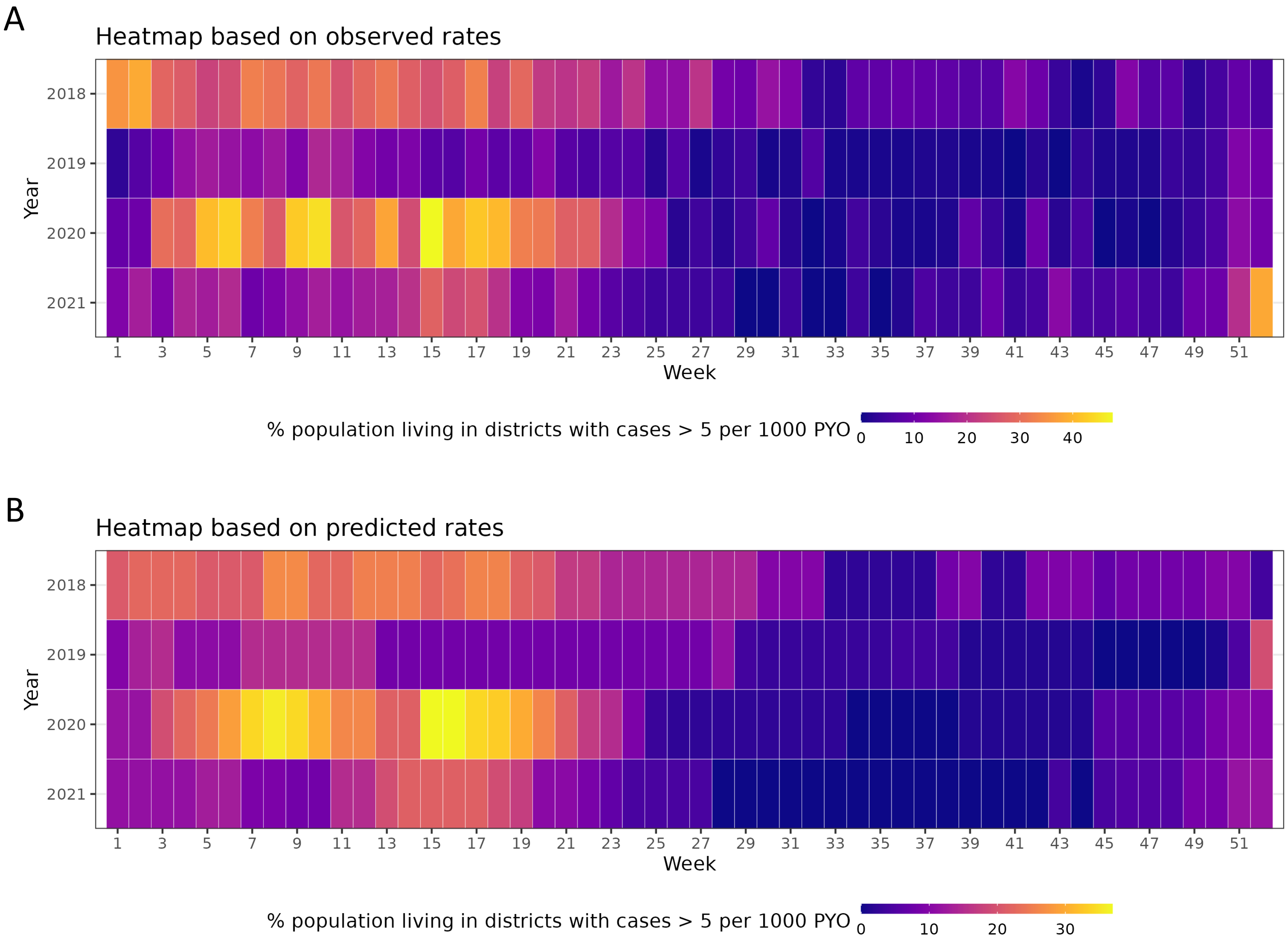

Supplement: Supplementary file 8 — Additional file 8. Heatmaps of A observed and B predicted rates showing the weekly percentage of population living in districts with over 5 cases per 1000 PYO from 2018 to 2021. [file 12936_2024_5103_MOESM8_ESM.pdf]
